# Meeting the 24-h movement recommendations and its relationship with Mediterranean dietary patterns in early childhood: the SENDO project

**DOI:** 10.1007/s00431-024-05472-z

**Published:** 2024-03-02

**Authors:** José Francisco López-Gil, Elise Fabios, Nerea Martín-Calvo

**Affiliations:** 1 One Health Research Group, Universidad de Las Américas, Quito, Ecuador; 2 Department of Preventive Medicine and Public Health, Facultad de Medicina, Universidad de Navarra, 31008 Pamplona, Spain; 3 IdiSNA, Instituto de Investigación Sanitaria de Navarra, 31008 Pamplona, Spain; 4 Pathophysiology of Obesity and Nutrition, Centro de Investigación Biomédica en Red, Instituto de Salud Carlos III, 28029 Madrid, Spain

**Keywords:** Physical activity, Sedentary behavior, Screen time, Sleep duration, Mediterranean diet, Preschoolers

## Abstract

The aim of this study was twofold: (1) to assess the association between meeting all three 24-h movement recommendations and adherence to the Mediterranean diet (MedDiet) in early childhood and (2) to examine whether participants who meet all three 24-h movement recommendations have greater adherence to the individual MedDiet foods/components than those who do not meet these recommendations. A cross-sectional study was conducted using a sample of 822 participants from the *Seguimiento del Niño para un Desarrollo* *Óptimo* (SENDO) project (Pamplona, Spain). Physical activity was assessed through a questionnaire that covered 17 different types of activities. Screen time was assessed by averaging the daily hours spent on activities such as watching TV, using a computer, or playing video games. Sleep duration was determined by taking the average of sleep durations during both weekdays and weekends. Adherence to the MedDiet was evaluated using the Mediterranean Diet Quality Index in children and adolescents (KIDMED). Greater odds of having an optimal adherence to the MedDiet were found for participants meeting all three 24-h movement recommendations (odds ratio (OR) = 1.96, 95% confidence interval (CI) 1.33–2.87, *p* = 0.001) in comparison with their counterparts not meeting these recommendations. Specifically, significant differences were found for “fruit or fruit juice every day” (*p* = 0.012), “second fruit every day” (*p* = 0.001), and “fresh or cooked vegetables regularly once a day” (*p* = 0.018) in relation to meeting all three 24-h movement recommendation status.

*    Conclusions*: This study provides further evidence of the potential importance of meeting all three 24-h movement recommendations to adopt a healthier eating pattern.

**Table:** 

**What is Known:** *• Only a limited number of studies have investigated the joint connection between the 24-h movement recommendations and dietary habits.* *• Integrating the 24-h movement recommendations with dietary recommendations could potentially yield greater effectiveness compared to promoting these recommendations independently within public health strategies.*	
**What is New:** *• This is the first study in assessing the relationship between 24-h movement recommendations and adherence to the Mediterranean diet in early childhood.* *• These findings provide further evidence of the potential importance of meeting all three 24-h movement recommendations to adopt a healthier eating pattern.*

**Supplementary Information:**

The online version contains supplementary material available at 10.1007/s00431-024-05472-z.

## Introduction

The Mediterranean diet (MedDiet) is the traditional eating style in nations bordering the Mediterranean Sea [1]. It features substantial consumption of fruits, vegetables, whole-grain bread, rice, legumes, and nuts, with moderate intakes of fish, dairy (especially cheese and yogurt), and restricted red meat and saturated fats [2]. Scientific literature links MedDiet adherence to reduced non-communicable disease occurrence in adults, including cancer, metabolic syndrome, hypertension, and cardiovascular diseases [3]. In young individuals, MedDiet adherence is associated with anti-inflammatory benefits [4], higher health-related quality of life [5], lower depression symptoms [6], cognitive performance [7], physical fitness [8], and reduced body mass index and obesity risk [9]. Despite these benefits, about half of European children and adolescents show limited adherence to the MedDiet [10].

Several factors may influence young individuals’ adherence to the MedDiet, with embracing a healthy lifestyle recognized as a relevant factor [11]. In this context, the 24-h movement recommendations for physical activity, sedentary behavior, and sleep duration have shifted the focus from individual elements to the incorporation of all movement-related pursuits throughout the entire day [12]. Numerous health and developmental outcomes have been linked to physical activity, sleep duration, and sedentary behaviors (e.g., screen time) among young people [13, 14]. While most studies have investigated these behaviors in isolation, there is a recent shift toward a more unified approach, considering the interconnected and mutually dependent nature of 24-h movement behaviors [15, 16]. Addressing these behaviors collectively aims to enhance health outcomes, such as reducing the risk of depression, obesity, and type 2 diabetes [13, 15, 17–19]. Despite the claimed health advantages, a recent meta-analysis revealed that only 7.1% of young people worldwide followed the 24-h movement recommendations [20].

To date, only a limited number of studies have explored the joint connection between the 24-h movement recommendations and dietary habits [21–23], with only one specifically investigating this association with adherence to the MedDiet in adolescents [21]. For instance, Thivel et al. [22] observed that children meeting all three 24-h movement recommendations reported a healthier dietary pattern compared to those not meeting any of these three recommendations. Specifically, Tapia-Serrano et al. [21] observed that adolescents meeting all three 24-h movement recommendations had higher odds of optimal adherence to the MedDiet; increased consumption of fruit/fruit juice, vegetables, fish, and cereals or grains (for breakfast); and lower consumption of commercially baked goods or pastries (for breakfast) and sweets and candy. Despite these studies, there is currently no research examining this relationship in early childhood.

Integrating the 24-h movement recommendations with dietary guidelines could potentially enhance effectiveness compared to promoting these recommendations independently in public health strategies [24]. Thus, educators and health professionals might find the potential connection between the 24-h recommendations and various components of the MedDiet noteworthy for upcoming nutritional interventions. Therefore, this study aims to achieve two objectives: (1) assess the association between meeting all three 24-h movement recommendations and adherence to the MedDiet in early childhood and (2) examine whether participants meeting all three 24-h movement recommendations demonstrate greater adherence to individual MedDiet foods/components than those not meeting these recommendations.

## Materials and methods

### Study population

The ongoing Spanish pediatric prospective cohort, known as the *Seguimiento del Niño para un Desarrollo* *Óptimo* (SENDO) project, aims to comprehend how children’s health is influenced by their diet and lifestyle. This multipurpose study focuses on preventing non-communicable diseases, specifically investigating the effects of the Mediterranean food pattern within a Mediterranean context, particularly in Spain.

To be eligible for participation, individuals must fall within the age range of 4 to 5 years at the time of recruitment and reside in Spain. Data collection occurs at the beginning and is updated annually through self-administered online surveys primarily completed by parents or legal guardians. The initial questionnaire (referred to as Q_0_) gathers comprehensive details about participants’ personal and family backgrounds, sociodemographic circumstances, physical measurements, dietary habits, eating patterns, lifestyles, physical activity levels, and personality characteristics. The collected data are inputted electronically and transferred to a secure online database.

From the initial 990 participants (100%) in the SENDO cohort, this cross-sectional analysis focuses on a sample of 822 participants (83%), excluding those without the variables of interest for this study. Figure S1 provides detailed information on the complete selection process.

### Procedures

#### 24-h movement recommendations (independent variable)

The 24-h movement recommendations for the age phase covered in this study are outlined as follows [12, 25]:

- Physical activity:
  Children under 5 years old should engage in a mix of physical activities throughout the day, totaling at least 180 min, with a minimum of 60 min involving vigorous play.
  Children 5 years old and older should accumulate a minimum of 60 min daily in moderate to vigorous physical activities, encompassing diverse aerobic exercises.
- Recreational screen time:
  Children under five should limit sedentary screen time to 1 h or less per day.
  Children 5 years old and older should restrict recreational screen time to a maximum of 2 h each day.
- Sleep duration:
  Children under 5 years need approximately 10 to 13 h of high-quality sleep, including the possibility of a nap. Maintaining consistent sleep and wake schedules is important.
  Children 5 years old and older should aim for uninterrupted sleep lasting 9 to 11 h per night.

The study collected information about physical activity through a questionnaire covering 17 different types of activities and 10 response options, ranging from never to over 10 h per week. The Spanish version of the questionnaire was validated in the “*Seguimiento Universidad de Navarr*a” study [26]. To calculate the metabolic equivalents of task (METs) per week for each activity, researchers multiplied the MET value of each activity by the weekly frequency of engagement, adjusting for the number of months spent on each activity [27]. Activities exceeding > 5 METs were considered vigorous, while those ≤ 5 METs were considered moderate. Total physical activity was determined by summing the MET-hours per week for activities performed during leisure time [28]. Additionally, screen time was assessed by averaging the daily hours spent on activities such as watching TV, using a computer, or playing video games. Overall sleep duration was determined by averaging sleep durations during both weekdays and weekends, calculated using the formula [(average sleep duration on weekdays × 5) + (average sleep duration on weekends × 2)] divided by 7.

#### Mediterranean diet (dependent variable)

Adherence to the MedDiet was evaluated using the Mediterranean Diet Quality Index in children and adolescents (KIDMED) [29], which is the most commonly used tool for measuring adherence to the MedDiet in young populations. The KIDMED consists of 16 items, 12 positive items (score 0 or + 1), and four negative items (score − 1 or 0), to evaluate the consumption of different food groups and components of the MedDiet. Thus, the final score ranged from − 4 to 12 points. Furthermore, participants were considered to have low adherence (≤ 3 points), moderate adherence (4–7 points), or high adherence (≥ 8 points) to the MedDiet. For our purposes, we grouped these categories into non-optimal adherence (< 8 points) and optimal adherence (≥ 8 points) to the MedDiet.

#### Covariates

Participants’ sex, age, weight, and height were reported by parents/guardians. The body mass index was calculated by dividing the weight in kilograms by the height in meters squared and, therefore, body mass index *z*-scores were obtained using the World Health Organization criteria [30]. Mother’s educational level and number of siblings in the household were self-reported by parents/guardians. Participants’ body mass index was calculated by dividing body weight in kilograms (kg) by height in meters squared (m^2^). A very high concordance was found between the anthropometric information declared by the parents and the direct measurement in this cohort [31]. Finally, the proportion of participants who took regular medication including treatment for asthma, allergy, attention deficit and hyperactivity disorder, constipation, immune system stimulant, or homeopathy was also reported.

### Statistical analysis

The main characteristics of the study’s participants were presented based on their adherence to the 24-h movement recommendations. Normality of the variables was assessed using visual methods (density and quantile–quantile plots) and Shapiro–Wilk’s test. For continuous variables, median and interquartile range were reported, while counts and percentages were provided for categorical variables. Associations between study variables and meeting all three 24-h movement recommendations were tested using the Mann–Whitney *U* test (for continuous variables) or chi-square test (for categorical variables). To examine the association between 24-h movement recommendations and adherence to the MedDiet, generalized linear models (GLMs) and generalized estimating equations (GEEs) were employed. GEEs extended GLMs to account for correlated observations. ORs and 95% confidence intervals (CIs) for optimal adherence to the MedDiet were calculated using the category “not meeting all three recommendations” as the reference. Analyses were presented unadjusted and adjusted for sex, age, parents’ educational level, body mass index, and the presence of siblings. Predictive margins and average marginal effects of meeting specific items of the KIDMED were also determined based on adherence to all three 24-h movement recommendations. To ensure comparability, analyses were further adjusted by the weighted KIDMED score, considering the exclusion of the item analyzed as a dependent variable in each analysis. All statistical analyses were conducted using R (Version 4.3.2) and RStudio (Version 2023.09.1 + 494), with a significance level set at *p* < 0.05.

## Results

Table 1 shows the main characteristics of the study participants. Overall, 21% of the participants examined met all three 24-h movement recommendations. The proportion of participants with optimal adherence to the MedDiet was 43%. According to the 24-h movement recommendation status, the proportion of participants who had an optimal adherence to the MedDiet was higher in those who met all three 24-h movement recommendations (54%) than in those who did not (40%).

**Table 1:** Descriptive data of the study participants in the SENDO project enrolled between January 2015 and June 2023 (*N* = 822)

	Meeting 24-h movement recommendations		
Variables	Not meeting all three (*n* = 652 (79%))	Meeting all three (*n* = 170 (21%))	*p* value
Age (years)	4.5 (1.0)	5.5 (0.5)	< 0.001
Sex			
Boys (%)	331 (51)	95 (56)	0.270
Girls (%)	321 (49)	75 (44)	
Weight (kg)	18.0 (3.1)	20.0 (3.7)	< 0.001
Height (cm)	109.0 (8.0)	114.0 (8.8)	< 0.001
Body mass index (kg/m^2^)	15.4 (1.9)	15.5 (1.9)	0.896
Body mass index (*z*-score)^a^	0.2 (1.4)	0.2 (1.3)	0.874
Mother’s educational level			
Non-university studies (%)	143 (22)	25 (15)	0.048
University studies (%)	509 (78)	145 (85)	
KIDMED (score)	7.0 (3.0)	8.0 (2.8)	< 0.001
Regular medication^b^			
No (%)	417 (64)	117 (69)	0.274
Yes (%)	235 (36)	53 (31)	
Adherence to the MedDiet (status)			
Non-optimal (%)	390 (60)	79 (47)	0.002
Optimal (%)	262 (40)	91 (55)	

Data presented as median (interquartile range) or count (percentage)

*KIDMED* Mediterranean Diet Quality Index in children and adolescents, *MedDiet* Mediterranean diet

^a^According to the World Health Organization criteria [30]

^b^Including treatment for asthma, allergy, attention deficit and hyperactivity disorder, constipation, immune system stimulant, or homeopathy

Table 2 depicts the unadjusted and adjusted odds ratios of having optimal adherence to the MedDiet according to meeting all three 24-h movement recommendation status. After adjusting for several covariates and considering the presence of siblings, greater odds of having an optimal adherence to the MedDiet were found for participants meeting all three 24-h movement recommendations (OR = 1.96, 95% CI 1.33–2.87, *p* = 0.001) in comparison with their counterparts not meeting these recommendations. Table S1 shows a significant linear trend between the number of 24-h movement recommendations met and the odds of having an optimal adherence to the MedDiet. Further detail on this association can be found in Table S1.

**Table 2:** Odds of having an optimal adherence to the Mediterranean diet based on the number of 24-h movement recommendations met in early childhood

	Optimal MedDiet adherence	
	*Unadjusted*	*Adjusted*
24-h movement recommendations	OR (95% CI)	OR (95% CI)^a,b^
Not meeting all three	Reference	Reference
Meeting all three	1.71 (1.22–2.41, *p* = 0.002)	1.96 (1.33–2.87, *p* = 0.001)

*CI* confidence interval, *MedDiet* Mediterranean diet, *OR* odds ratio

^a^Adjusted for age, sex, mother’s educational level, and body mass index

^b^Considering the presence of siblings in the household

Table 3 indicates the predictive margins and average marginal effects of reporting each specific KIDMED item based on meeting all three 24-h movement recommendation status. Significant differences were found for item 1 (“Takes a fruit or fruit juice every day”) (*p* = 0.012), item 2 (“Has a second fruit every day”) (*p* = 0.001), and item 3 (“Has fresh or cooked vegetables regularly once a day”) (*p* = 0.018). For item 4 (“Consumes nuts regularly (at least 2–3 times per week)”), a borderline significant association was observed (*p* = 0.082). Additional information reporting the predictive margins based on the number of 24-h movement recommendations met can be found in Table S2.

**Table 3:** Predictive margins^a^ and average marginal effects^b^ of meeting all three 24-h movement recommendations based on each specific item of the Mediterranean Diet Quality Index in children and adolescents

	Meeting all three 24-h movement recommendations			
	Not meeting	Meeting		
KIDMED items (dependent variable)	PM (95% CI)	PM (95% CI)	AME	*p*
Takes a fruit or fruit juice every day^c^	94 (92, 95)	98 (95, 100)	4.3 (0.9, 7.6)	**0.012**
Has a second fruit every day^c^	65 (61, 68)	78 (71, 85)	13 (5.2, 21)	**0.001**
Has fresh or cooked vegetables regularly once a day^c^	74 (71, 77)	83 (77, 89)	8.4 (1.5, 15)	**0.018**
Has fresh or cooked vegetables more than once a day^c^	36 (32, 39)	42 (35, 50)	6.9 (− 1.7, 16)	0.115
Consumes fish regularly (at least 2–3 times per week)^c^	77 (74, 81)	83 (77, 89)	5.4 (− 1.4, 12)	0.119
Goes more than once a week to a fast-food (hamburger) restaurant^‡^	1.0 (0.2, 1.7)	1.8 (− 1.1, 4.7)	0.8 (− 2.2, 3.8)	0.586
Likes pulses and eats them more than once a week^c^	94 (93, 96)	95 (91, 99)	0.7 (− 3.7, 5.2)	0.747
Consumes pasta or rice almost every day (5 or more times per week)^c^	29 (25, 32)	34 (26, 42)	4.9 (− 4.1, 14)	0.288
Has cereals or grains (bread, etc.) for breakfast^c^	66 (63, 70)	64 (56, 72)	− 2.0 (− 11, 6.9)	0.655
Consumes nuts regularly (at least 2–3 times per week)^c^	24 (21, 28)	32 (24, 39)	7.4 (− 9.5, 16)	0.082
Uses olive oil at home^c^	68 (65, 72)	72 (65, 79)	3.6 (− 4.0, 11)	0.352
Skips breakfast^d^	3.7 (2.1, 5.2)	5.7 (1.4, 10)	2.3 (− 3.2, 7.9)	0.405
Has a dairy product for breakfast (yogurt, milk, etc.)^c^	82.3 (79, 85)	85 (80, 91)	3.1 (− 3.8, 10)	0.377
Has commercially baked goods or pastries for breakfast^d^	31.9 (28, 36)	28 (20, 35)	− 4.3 (− 13, 4.1)	0.320
Takes two yogurts and/or some cheese (40 g) daily^c^	17 (14, 20)	21 (14, 27)	3.3 (− 4.2, 11)	0.385
Takes sweets and candy several times every day^d^	8.0 (5.9, 10)	12 (6.4, 17)	3.7 (− 2.2, 9.7)	0.219

AME represents the expected change in the dependent variable for a one-unit change in an independent variable (i.e., from non-meeting with all three 24-h movement recommendations to meeting with all three 24-h movement recommendations)

Bold indicates a *p* value < 0.05

*AME* average marginal effects, *CI* confidence interval, *KIDMED* Mediterranean Diet Quality Index in children and adolescents, *PM* predictive margin

^a^Predictive margin (i.e., probability) of meeting all three 24-h movement recommendations for each KIDMED item after adjusting for age, sex, mother’s educational level, and body mass index (considering the presence of siblings)

^b^Average marginal effects (i.e., the average of predicted differences in fitted values for meeting all three 24-h movement recommendation status)

^c^Further adjusted for KIDMED score multiplied by the weighting of the positive items (+ 1) to adjust the point scale after eliminating one of them (12/11)

^d^Further adjusted for KIDMED score multiplied by the weighting of the negative items (− 1) to adjust the point scale after eliminating one of them (4/3)

## Discussion

This study represents the first exploration of the relationship between 24-h movement recommendations and adherence to the MedDiet in early childhood. In summary, our results suggest that children adhering to all three recommendations were more likely to have a higher KIDMED score and follow the MedDiet compared to those not meeting these recommendations. Specifically, higher odds were observed for consuming one piece of fruit or fruit juice daily, a second piece of fruit daily, and fresh or cooked vegetables among those meeting all three 24-h movement recommendations, in contrast to their counterparts not meeting these recommendations. These findings align with previous research conducted in different populations, including children [22, 32] and adolescents [21, 23], as well as a review by Fournier et al. [24], which advocates for the integration of nutritional recommendations within the framework of 24-h movement recommendations. Notably, while such associations have been explored in older age groups, this study is the first to investigate this relationship in early childhood. Several potential reasons may account for these findings.

One possible explanation for the observed relationship between adherence to 24-h movement recommendations and adherence to the MedDiet may be attributed to the phenomenon of clustering among healthy and unhealthy behaviors [33, 34]. The concept of clustering is grounded in the understanding that lifestyle influences are diverse and interconnected [33]. In line with this concept, a previous review highlighted the close relationship and interconnected nature of 24-h movement behaviors and dietary patterns [24]. The adoption of healthier behaviors can trigger a ripple effect, leading to the establishment of other beneficial habits. Conversely, unhealthy behaviors tend to cluster, forming a cycle that is challenging to break [24, 35]. Indeed, a systematic review and meta-analysis reported that children and adolescents with higher adherence to the MedDiet tend to engage in higher levels of physical activity, exhibit greater physical fitness, and participate in less sedentary lifestyles [34]. Engaging in physical activity can serve as a pathway to adopting other positive habits, as knowledge, experiences, skills, and self-confidence gained from one behavior can transfer and influence the adoption of others [36]. Regular physical activity can assist in establishing a routine and forming healthy habits. Once a routine is established, individuals may find it easier to incorporate other healthy behaviors, such as maintaining a Mediterranean-style diet [37]. Likewise, engaging in one positive behavior, like regular physical activity, can lead to positive reinforcement and a sense of well-being [38]. This positive reinforcement may motivate individuals to adopt other healthy habits, including maintaining a balanced and nutritious diet [39]. Success in one health behavior can boost an individual’s self-efficacy in their ability to make positive changes, which may spill over into other areas of life, making it more likely for individuals to adopt and sustain healthy behaviors [40]. These factors collectively provide possible explanations for the observed dose–response relationship in our analyses.

Another potential reason for the observed relationship may be linked to the role of appetite control. A review by Julian et al. [41] highlighted that studies investigating energy balance regulation suggest that favorable movement behavior profiles are associated with healthier eating habits and enhanced appetite control. Moreover, excessive TV viewing among young individuals has been associated with increased food consumption, especially of more palatable and less satiating foods like high-calorie snacks and sugar-sweetened beverages. This phenomenon is attributed to the “mindless eating” hypothesis [42, 43]. According to this hypothesis, when children and adolescents spend extended periods in front of screens, they may become less attentive to their eating habits as they engage in other activities simultaneously [44]. Furthermore, the role of sleep duration could be another contributing factor. A meta-analysis indicated that short sleep duration was specifically linked to unhealthier eating habits, such as lower fruit/vegetable consumption [45]. It is possible that sleep restriction may promote hedonic stimuli in the brain, altering brain connectivity and increasing the reward value of food [46], especially unhealthy foods [47]. Consistent with this, a recent review suggested that while the evidence on the association between sleep duration and levels of appetite-regulating hormones in adolescents is conflicting, insufficient sleep may impact dietary choices by enhancing the reward value of foods, particularly those high in carbohydrates [48].

Moreover, it is possible that engaging in physical activity might stimulate hunger, potentially leading to an increased intake of food to compensate for the energy expended during the activity [49]. Notably, engaging in physical activity before a meal has been associated with a reduction in immediate energy intake, especially when there is a shorter time gap between exercise and eating [50]. Additionally, inadequate sleep duration has been linked to a lower basal metabolic rate, reduced physical activity, and increased sedentary behaviors among young children [51]. Therefore, a hypothesis emerges that young children adhering to 24-h movement recommendations for physical activity, recreational screen time, and sleep duration during early childhood may maintain a more active lifestyle, possibly requiring higher energy expenditure for their daily activities. In this context, fruits, rich in carbohydrates, and nuts, abundant in fats, could serve as valuable sources of fuel to meet the elevated energy demands associated with an active lifestyle.

Another possible explanation may be found in the role of essential nutrients. As mentioned earlier, individuals who are physically active may have increased energy demands, leading to a greater need for essential and non-essential nutrients such as vitamins and minerals. This heightened nutritional requirement could serve as a motivation for them to adopt healthier eating habits. For example, vitamin B6 is crucial for the proper release of glycogen stored in muscles and the liver, ensuring a continuous supply of energy during exercise. Fruits and vegetables, as components of the MedDiet, could contribute to a higher fiber intake [52]. It is conceivable that individuals meeting 24-h movement recommendations may have a greater fiber intake, aligning with findings from a previous study in children [32]. Considering an active lifestyle’s higher energy and fiber demands to support proper digestive system function [53], a diet rich in fruits and vegetables becomes beneficial. Conversely, a diet rich in ultra-processed foods, deviating from the main components of the MedDiet, might contribute to insufficiency of certain micronutrients [54]. Therefore, it is possible that active children may require increased amounts of vitamins (e.g., vitamin C and vitamin B6) and fiber, and adherence to the MedDiet (including fruits and vegetables) could, at least partially, explain this observed association.

This study has several limitations that should be acknowledged. First, the cross-sectional design impedes the establishment of causal relationships. The relationship could be bidirectional, and future longitudinal and experimental studies are needed to discern the directionality of this association. Second, although the questionnaires used to assess movement behaviors and diet are considered valid and reliable, reliance on parent-reported data may introduce a potential for overestimation of results [55]. Future research could enhance accuracy by incorporating device-based measurements for physical activity, sedentary behavior, and sleep duration. Third, the study did not assess parenting practices related to meal preparation and food selection, which is important given that participants in this age phase rely on meals prepared by their parents. Caution should be exercised in interpreting the results in this context. Fourth, the high proportion of mothers with university studies in both groups may limit the generalizability of findings to families with lower education levels, underscoring the need for more diverse educational level representation in future research. Finally, the observational design introduces the possibility of residual confounding by unaccounted variables. Despite these limitations, the study has notable strengths, including a substantial sample size. Analyses were adjusted for relevant factors such as age, sex, parents’ education level, and body mass index, and the presence of siblings in the household was considered through GEE models, enhancing the robustness of the results. The calculation of average marginal effects provides an intuitive technique for interpreting regression estimates, facilitating comparisons across studies [56].

In conclusion, this study contributes additional evidence supporting the potential significance of meeting all three 24-h movement recommendations for adopting a healthier eating pattern. The observed association between adherence to 24-h movement recommendations and adherence to MedDiet in early childhood suggests that promoting these movement behaviors could enhance the beneficial effects of the MedDiet on health.

### Supplementary Information

Below is the link to the electronic supplementary material.

Supplementary file1 (TIFF 67 KB)

Supplementary file2 (DOCX 23 KB)

## Data Availability

Data will be available upon a reasonable request.
