# Supplementary material for: Meeting the 24-h movement recommendations and its relationship with Mediterranean dietary patterns in early childhood: the SENDO project
Source: Eur J Pediatr. 2024 Mar 2;183(5):2365–73. doi: 10.1007/s00431-024-05472-z (PMC11035442; doi:10.1007/s00431-024-05472-z)
Supplement: Supplementary file 2 — Supplementary file2 (DOCX 23 KB) [file 431_2024_5472_MOESM2_ESM.docx]

**Supplementary material**

## Table SI. Odds of having an optimal adherence to the Mediterranean diet based on the number of 24-hour movement recommendations met in early childhood.

|  | Optimal MedDiet adherence | | | |
| --- | --- | --- | --- | --- |
|  | *Unadjusted* |  | *Adjusted* |  |
| 24-hour movement recommendations | OR (95% CI) | *p*-for-trend | OR (95% CI) ^†,‡^ | *p*-for-trend |
| No recommendation | Reference | 0.002 | Reference | <0.001 |
| One recommendation | 1.70 (0.96-3.01, *p*=0.069) |  | 1.79 (1.01-3.20, *p*=0.047) |  |
| Two recommendations | 2.00 (1.14-3.51, *p*=0.015) |  | 2.48 (1.37-4.47, *p*=0.003) |  |
| Three recommendations | 2.99 (1.64-5.44, *p<*0.001) |  | 4.34 (2.22-8.48, *p<*0.001) |  |

^†^ Adjusted for age, sex, mother’s educational level, and body mass index. MedDiet, Mediterranean diet; CI, confidence interval. ^‡^ Considering the presence of siblings in the household.

**Table SII.** Predictive margins of the number of 24-hour movement recommendations met based on each specific item of the Mediterranean Diet Quality Index in children and adolescents.

|  | Meeting all three 24-hour movement recommendations | | | |
| --- | --- | --- | --- | --- |
|  | No recommendation  (n = 72 – 8.8%) | One recommendation  (n = 263 – 32.0%) | Two recommendations  (n = 317 – 38.6%) | Three recommendations  (n = 170 – 20.7%) |
| KIDMED items (dependent variable) | PM (95% CI) | PM (95% CI) | PM (95% CI) | PM (95% CI) |
| Takes a fruit or fruit juice every day^†^ | 90.7 (85.7, 95.7) | 93.9 (90.9, 96.8) | 95.1 (92.2, 98.1) | 98.3 (95.9, 100.1)^a,b^ |
| Has a second fruit every day^†^ | 66.8 (58.9, 74.6) | 65.4 (59.8, 71.1) | 62.8 (55.5, 70.1) | 77.0 (69.7, 84.3)^b,c^ |
| Has fresh or cooked vegetables regularly once a day^†^ | 71.8 (64.5, 79.1) | 73.9 (68.8, 78.9) | 75.9 (70.1, 81.8) | 83.4 (77.2, 89.5)^a,b^ |
| Has fresh or cooked vegetables more than once a day^†^ | 30.3 (22.7, 37.9) | 29.7 (24.3, 35.0) | 45.4 (37.7, 53.1)^a,b^ | 47.2 (39.0, 55.5)^a,b^ |
| Consumes fish regularly (at least 2 – 3 times per week)^†^ | 75.5 (67.7, 83.2) | 78.0 (72.7, 83.3) | 77.4 (71.5, 83.2) | 82.8 (76.8, 88.8) |
| Goes more than once a week to a fast-food (hamburger) restaurant^‡^ | 5.1 (-2.2, 12.5) | - | 0.4 (-0.4, 0.13) | 1.2 (-0.5, 2.9) |
| Likes pulses and eats them more than once a week^†^ | 92.5 (87.9, 97.1) | 95.7 (93.3, 98.0) | 93.8 (90.1, 97.6) | 95.0 (90.8, 99.3) |
| Consumes pasta or rice almost every day (5 or more times per week)^†^ | 37.5 (28.5, 46.6) | 33.3 (27.2, 39.5) | 19.7 (13.6, 25.8)^a,b^ | 29.0 (21.2, 36.8)^c^ |
| Has cereals or grains (bread, etc.) for breakfast^†^ | 61.0 (52.2, 69.8) | 65.5 (59.7, 71.6) | 69.9 (62.9, 76.8) | 66.1 (57.9, 74.3) |
| Consumes nuts regularly (at least 2 – 3 times per week)^†^ | 21.7 (14.2, 29.2) | 27.4 (21.7, 33.1) | 22.2 (15.8, 28.6) | 30.7 (22.8, 38.5) |
| Uses olive oil at home^†^ | 55.7 (48.0, 65.4) | 65.2 (59.3, 71.1) | 76.7 (71.0, 82.4)^a,b^ | 75.8 (69.4, 82.1)^a,b^ |
| Skips breakfast^‡^ | 7.7 (2.7, 12.7) | 4.5 (1.9, 7.1) | 1.9 (-0.1, 4.0)^a^ | 5.0 (0.9, 9.1) |
| Has a dairy product for breakfast (yogurt, milk, etc.)^†^ | 80.7 (73.3, 88.1) | 80.5 (75.3, 85.7) | 85.1 (79.6, 90.5) | 86.5 (80.6, 92.4) |
| Has commercially baked goods or pastries for breakfast^‡^ | 35.6 (26.9, 44.4) | 31.8 (25.8, 37.8) | 30.1 (22.9, 37.3) | 26.7 (19.1, 34.4) |
| Takes two yogurts and/or some cheese (40 g) daily^†^ | 25.9 (17.2, 34.5) | 13.7 (9.2, 18.1)^a^ | 17.0 (11.2, 22.8) | 19.9 (13.0, 26.8) |
| Takes sweets and candy several times every day^‡^ | 10.9 (4.8, 17.0) | 8.8 (5.1, 12.5) | 6.1 (2.9, 9.3) | 10.7 (5.6, 15.7) |

Data expressed as predictive margins (i.e., probability) with 95% confidence interval of the number of 24-hour movement recommendations met according to each KIDMED item. Adjusted for age, sex, mother’s educational level, and body mass index (considering the presence of siblings).

KIDMED, Mediterranean Diet Quality Index in children and adolescents.

^a^ Statistically significant differences with the "no recommendation" group for average marginal effects (*p* < 0.05).

^b^ Statistically significant differences with the "one recommendation" group for average marginal effects (*p* < 0.05).

^c^ Statistically significant differences with the "two recommendations" group for average marginal effects (*p* < 0.05).

† Further adjusted for KIDMED score multiplied by the weighting of the positive items (+1) to adjust the point scale after eliminating one of them (12/11).

‡ Further adjusted for KIDMED score multiplied by the weighting of the negative items (-1) to adjust the point scale after eliminating one of them (4/3).
